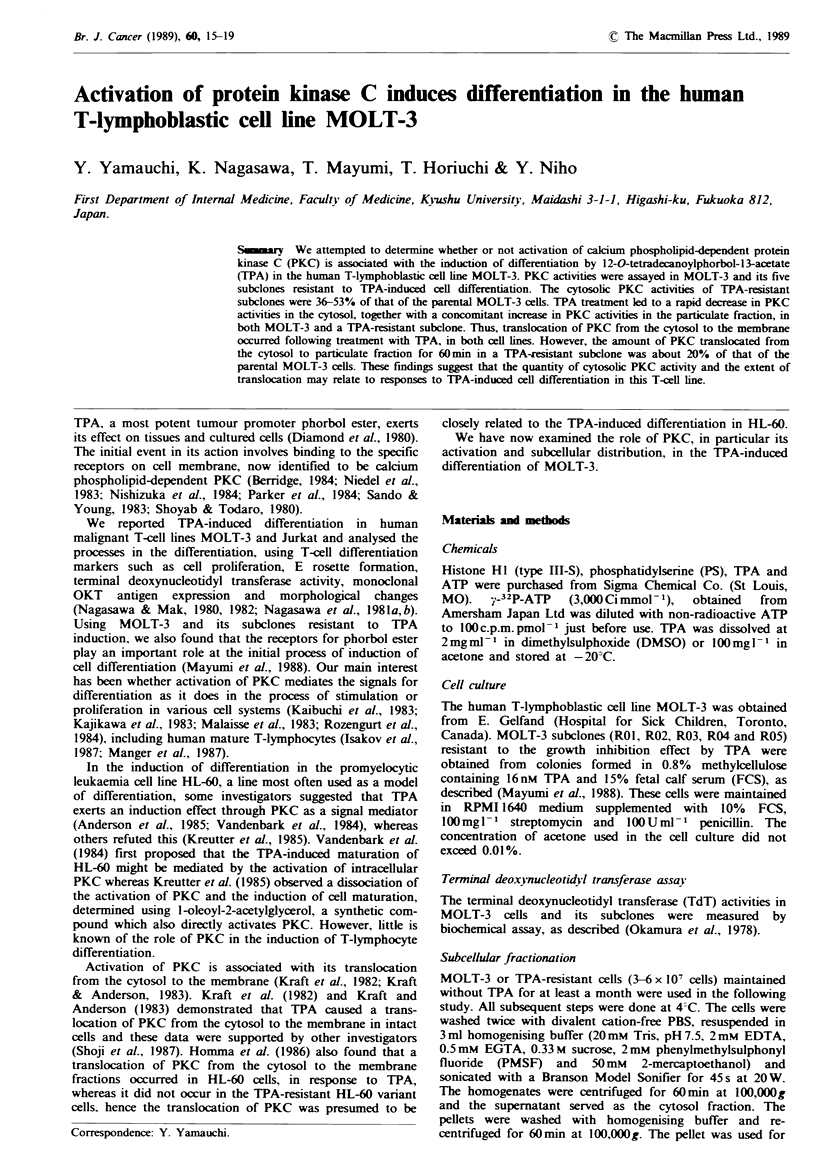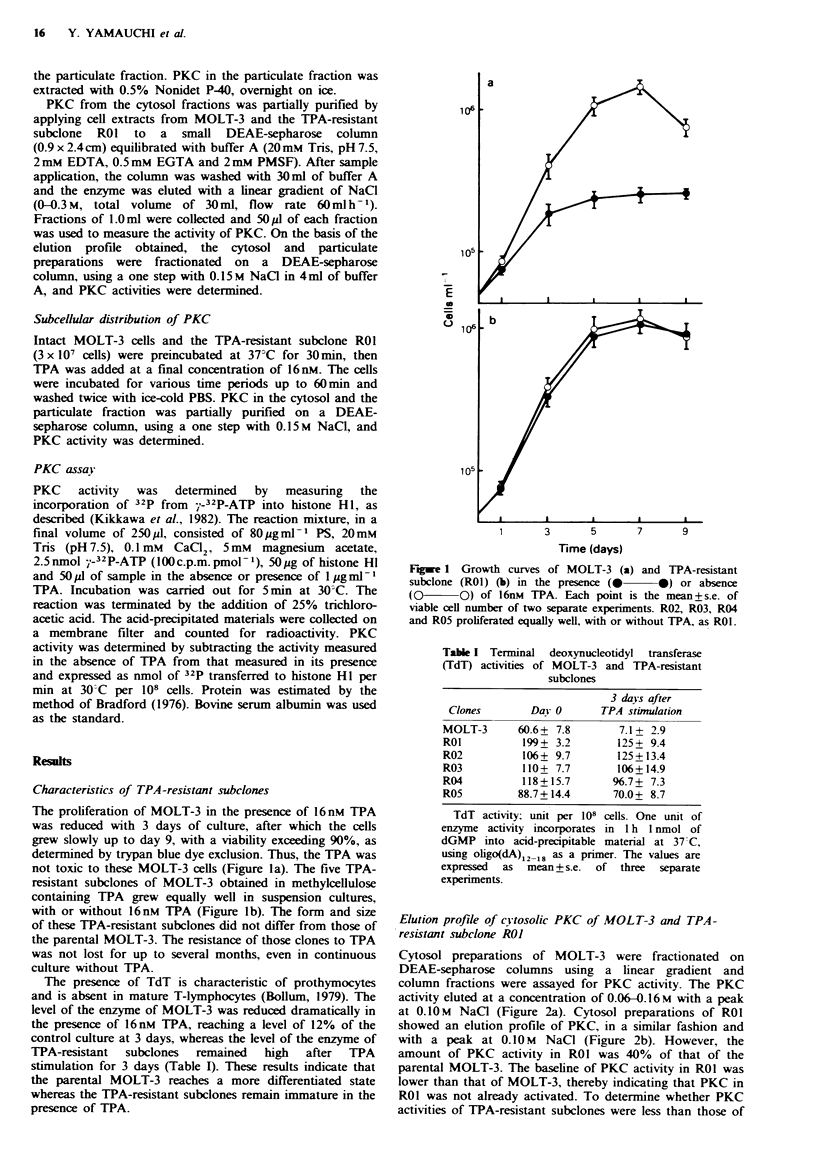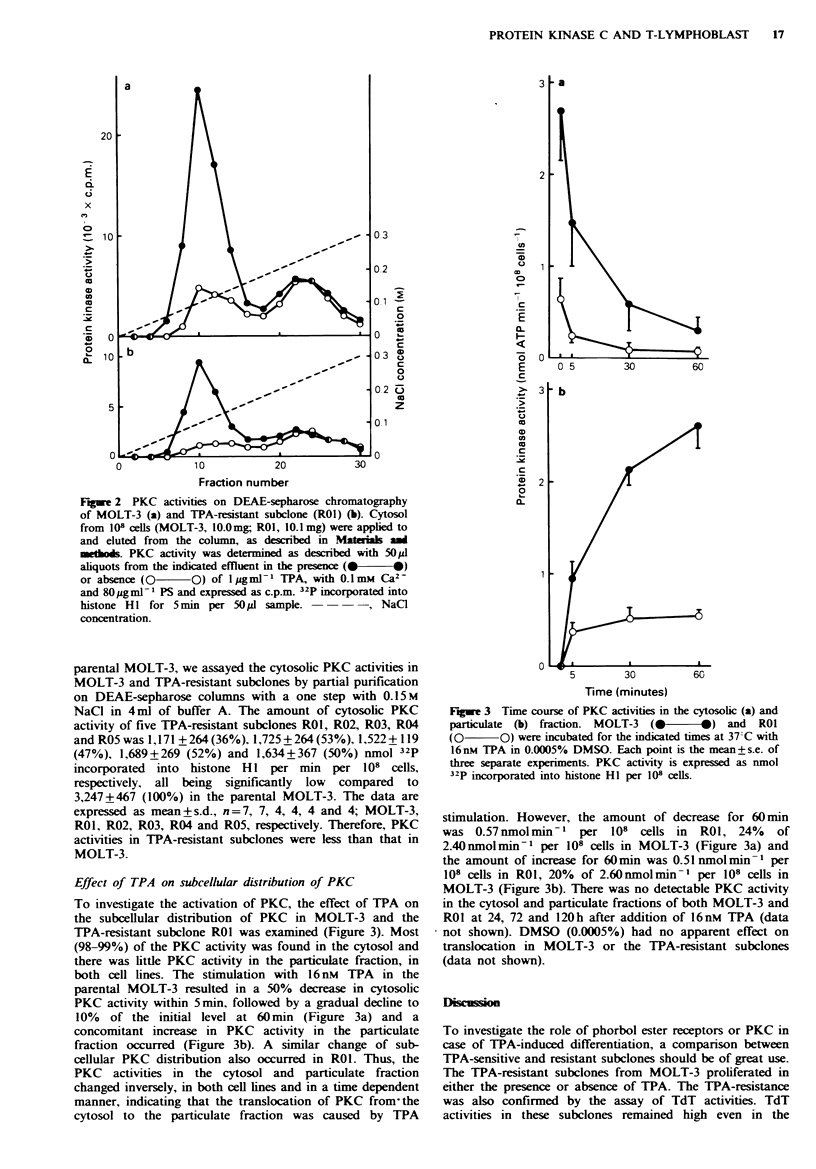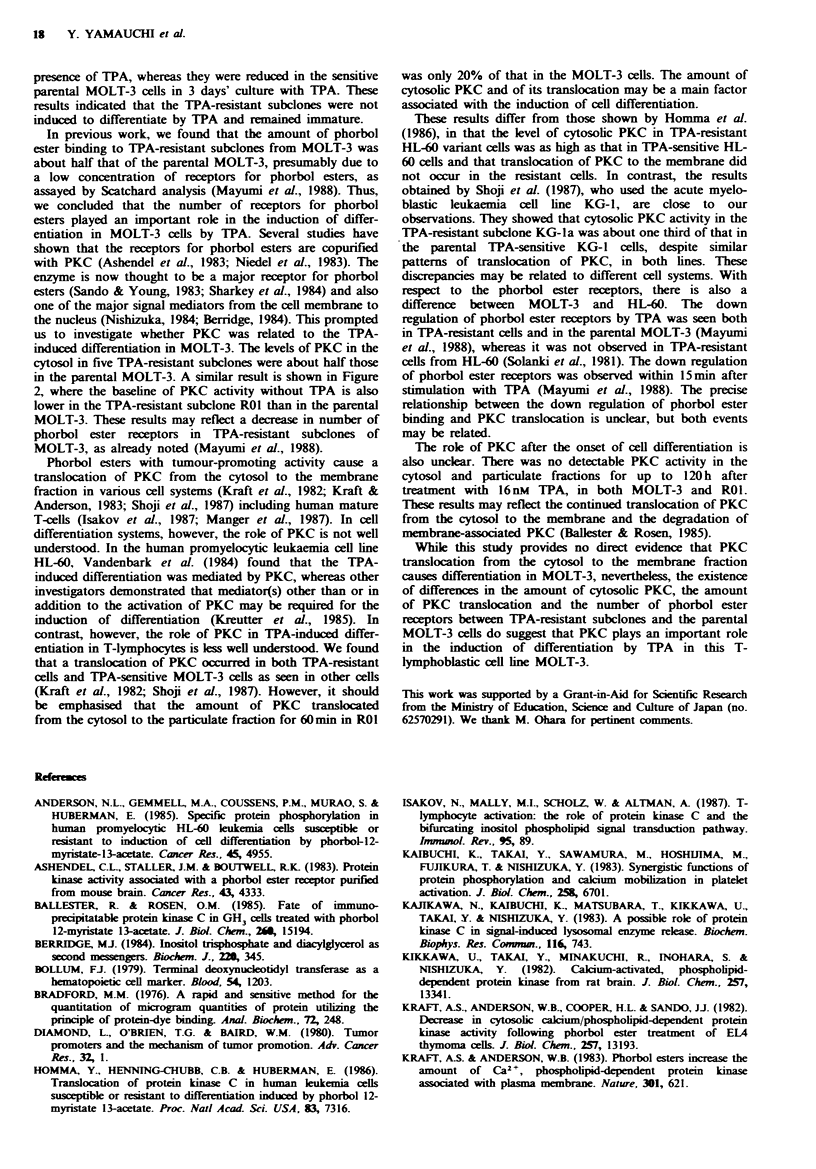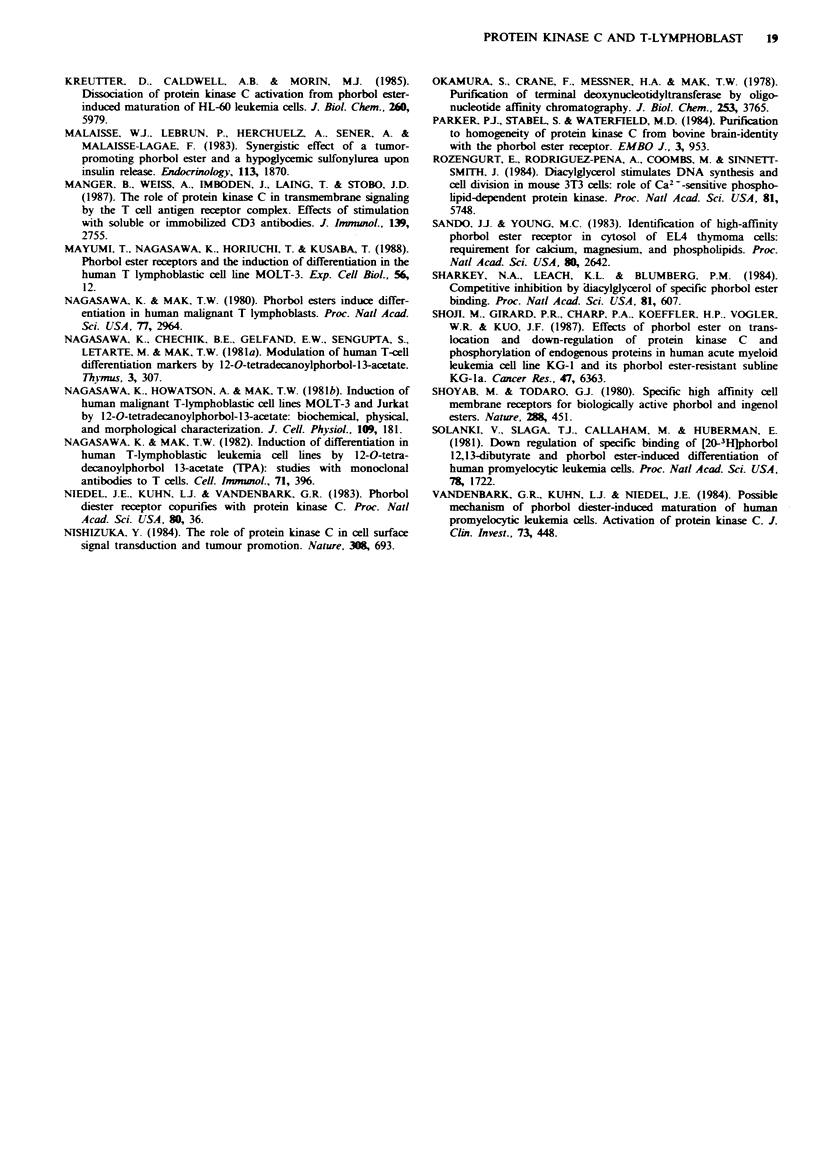# Activation of protein kinase C induces differentiation in the human T-lymphoblastic cell line MOLT-3.

**DOI:** 10.1038/bjc.1989.211

**Published:** 1989-07

**Authors:** Y. Yamauchi, K. Nagasawa, T. Mayumi, T. Horiuchi, Y. Niho

**Affiliations:** First Department of Internal Medicine, Faculty of Medicine, Kyushu University, Fukuoka, Japan.

## Abstract

We attempted to determine whether or not activation of calcium phospholipid-dependent protein kinase C (PKC) is associated with the induction of differentiation by 12-O-tetradecanoylphorbol-13-acetate (TPA) in the human T-lymphoblastic cell line MOLT-3. PKC activities were assayed in MOLT-3 and its five subclones resistant to TPA-induced cell differentiation. The cytosolic PKC activities of TPA-resistant subclones were 36-53% of that of the parental MOLT-3 cells. TPA treatment led to a rapid decrease in PKC activities in the cytosol, together with a concomitant increase in PKC activities in the particulate fraction, in both MOLT-3 and a TPA-resistant subclone. Thus, translocation of PKC from the cytosol to the membrane occurred following treatment with TPA, in both cell lines. However, the amount of PKC translocated from the cytosol to particulate fraction for 60 min in a TPA-resistant subclone was about 20% of that of the parental MOLT-3 cells. These findings suggest that the quantity of cytosolic PKC activity and the extent of translocation may relate to responses to TPA-induced cell differentiation in this T-cell line.


Br. J. Cancer (1989), 60, 15–19 © The Macmillan Press Ltd., 1989

### Activation of protein kinase C induces differentiation in the human T-lymphoblastic cell line MOLT-3

Y. Yamauchi, K. Nagasawa, T. Mayumi, T. Horiuchi & Y. Niho

*First Department of Internal Medicine, Faculty of Medicine, Kyushu University, Maidashi 3-1-1, Higashi-ku, Fukuoka 812, Japan.*

**Summary** We attempted to determine whether or not activation of calcium phospholipid-dependent protein kinase C (PKC) is associated with the induction of differentiation by 12-*O*-tetradecanoylphorbol-13-acetate (TPA) in the human T-lymphoblastic cell line MOLT-3. PKC activities were assayed in MOLT-3 and its five subclones resistant to TPA-induced cell differentiation. The cytosolic PKC activities of TPA-resistant subclones were 36–53% of that of the parental MOLT-3 cells. TPA treatment led to a rapid decrease in PKC activities in the cytosol, together with a concomitant increase in PKC activities in the particulate fraction, in both MOLT-3 and a TPA-resistant subclone. Thus, translocation of PKC from the cytosol to the membrane occurred following treatment with TPA, in both cell lines. However, the amount of PKC translocated from the cytosol to particulate fraction for 60 min in a TPA-resistant subclone was about 20% of that of the parental MOLT-3 cells. These findings suggest that the quantity of cytosolic PKC activity and the extent of translocation may relate to responses to TPA-induced cell differentiation in this T-cell line.

TPA, a most potent tumour promoter phorbol ester, exerts its effect on tissues and cultured cells (Diamond *et al.*, 1980). The initial event in its action involves binding to the specific receptors on cell membrane, now identified to be calcium phospholipid-dependent PKC (Berridge, 1984; Niedel *et al.*, 1983; Nishizuka *et al.*, 1984; Parker *et al.*, 1984; Sando & Young, 1983; Shoyab & Todaro, 1980).

We reported TPA-induced differentiation in human malignant T-cell lines MOLT-3 and Jurkat and analysed the processes in the differentiation, using T-cell differentiation markers such as cell proliferation, E rosette formation, terminal deoxynucleotidyl transferase activity, monoclonal OKT antigen expression and morphological changes (Nagasawa & Mak, 1980, 1982; Nagasawa *et al.*, 1981*a,b*). Using MOLT-3 and its subclones resistant to TPA induction, we also found that the receptors for phorbol ester play an important role at the initial process of induction of cell differentiation (Mayumi *et al.*, 1988). Our main interest has been whether activation of PKC mediates the signals for differentiation as it does in the process of stimulation or proliferation in various cell systems (Kaibuchi *et al.*, 1983; Kajikawa *et al.*, 1983; Malaisse *et al.*, 1983; Rozengurt *et al.*, 1984), including human mature T-lymphocytes (Isakov *et al.*, 1987; Manger *et al.*, 1987).

In the induction of differentiation in the promyelocytic leukaemia cell line HL-60, a line most often used as a model of differentiation, some investigators suggested that TPA exerts an induction effect through PKC as a signal mediator (Anderson *et al.*, 1985; Vandenbark *et al.*, 1984), whereas others refuted this (Kreutter *et al.*, 1985). Vandenbark *et al.* (1984) first proposed that the TPA-induced maturation of HL-60 might be mediated by the activation of intracellular PKC whereas Kreutter *et al.* (1985) observed a dissociation of the activation of PKC and the induction of cell maturation, determined using 1-oleoyl-2-acetylglycerol, a synthetic compound which also directly activates PKC. However, little is known of the role of PKC in the induction of T-lymphocyte differentiation.

Activation of PKC is associated with its translocation from the cytosol to the membrane (Kraft *et al.*, 1982; Kraft & Anderson, 1983). Kraft *et al.* (1982) and Kraft and Anderson (1983) demonstrated that TPA caused a translocation of PKC from the cytosol to the membrane in intact cells and these data were supported by other investigators (Shoji *et al.*, 1987). Homma *et al.* (1986) also found that a translocation of PKC from the cytosol to the membrane fractions occurred in HL-60 cells, in response to TPA, whereas it did not occur in the TPA-resistant HL-60 variant cells, hence the translocation of PKC was presumed to be closely related to the TPA-induced differentiation in HL-60.

We have now examined the role of PKC, in particular its activation and subcellular distribution, in the TPA-induced differentiation of MOLT-3.

### Materials and methods

### Chemicals

Histone H1 (type III-S), phosphatidylserine (PS), TPA and ATP were purchased from Sigma Chemical Co. (St Louis, MO). $\gamma$-$^{32}$P-ATP (3,000 Ci mmol$^{-1}$), obtained from Amersham Japan Ltd was diluted with non-radioactive ATP to 100 c.p.m. pmol$^{-1}$ just before use. TPA was dissolved at 2 mg ml$^{-1}$ in dimethylsulphoxide (DMSO) or 100 mg l$^{-1}$ in acetone and stored at $-20$°C.

### Cell culture

The human T-lymphoblastic cell line MOLT-3 was obtained from E. Gelfand (Hospital for Sick Children, Toronto, Canada). MOLT-3 subclones (R01, R02, R03, R04 and R05) resistant to the growth inhibition effect by TPA were obtained from colonies formed in 0.8% methylcellulose containing 16 nM TPA and 15% fetal calf serum (FCS), as described (Mayumi *et al.*, 1988). These cells were maintained in RPMI 1640 medium supplemented with 10% FCS, 100 mg l$^{-1}$ streptomycin and 100 U ml$^{-1}$ penicillin. The concentration of acetone used in the cell culture did not exceed 0.01%.

### Terminal deoxynucleotidyl transferase assay

The terminal deoxynucleotidyl transferase (TdT) activities in MOLT-3 cells and its subclones were measured by biochemical assay, as described (Okamura *et al.*, 1978).

### Subcellular fractionation

MOLT-3 or TPA-resistant cells ($3$–$6\times10^7$ cells) maintained without TPA for at least a month were used in the following study. All subsequent steps were done at 4°C. The cells were washed twice with divalent cation-free PBS, resuspended in 3 ml homogenising buffer (20 mM Tris, pH 7.5, 2 mM EDTA, 0.5 mM EGTA, 0.33 M sucrose, 2 mM phenylmethylsulphonyl fluoride (PMSF) and 50 mM 2-mercaptoethanol) and sonicated with a Branson Model Sonifier for 45 s at 20 W. The homogenates were centrifuged for 60 min at 100,000 *g* and the supernatant served as the cytosol fraction. The pellets were washed with homogenising buffer and re-centrifuged for 60 min at 100,000 *g*. The pellet was used for

Correspondence: Y. Yamauchi.

the particulate fraction. PKC in the particulate fraction was extracted with 0.5% Nonidet P-40, overnight on ice.

PKC from the cytosol fractions was partially purified by applying cell extracts from MOLT-3 and the TPA-resistant subclone R01 to a small DEAE-sepharose column (0.9 x 2.4 cm) equilibrated with buffer A (20 mM Tris, pH 7.5, 2 mM EDTA, 0.5 mM EGTA and 2 mM PMSF). After sample application, the column was washed with 30 ml of buffer A and the enzyme was eluted with a linear gradient of NaCl (0–0.3 M, total volume of 30 ml, flow rate 60 ml $h^{-1}$). Fractions of 1.0 ml were collected and 50 $\mu$l of each fraction was used to measure the activity of PKC. On the basis of the elution profile obtained, the cytosol and particulate preparations were fractionated on a DEAE-sepharose column, using a one step with 0.15 M NaCl in 4 ml of buffer A, and PKC activities were determined.

*Subcellular distribution of PKC*

Intact MOLT-3 cells and the TPA-resistant subclone R01 ($3 \times 10^7$ cells) were preincubated at 37°C for 30 min, then TPA was added at a final concentration of 16 nM. The cells were incubated for various time periods up to 60 min and washed twice with ice-cold PBS. PKC in the cytosol and the particulate fraction was partially purified on a DEAE-sepharose column, using a one step with 0.15 M NaCl, and PKC activity was determined.

*PKC assay*

PKC activity was determined by measuring the incorporation of $^{32}P$ from $\gamma$-$^{32}P$-ATP into histone H1, as described (Kikkawa *et al.*, 1982). The reaction mixture, in a final volume of 250 $\mu$l, consisted of 80 $\mu g\ ml^{-1}$ PS, 20 mM Tris (pH 7.5), 0.1 mM $CaCl_2$, 5 mM magnesium acetate, 2.5 nmol $\gamma$-$^{32}P$-ATP (100 c.p.m. $pmol^{-1}$), 50 $\mu$g of histone HI and 50 $\mu$l of sample in the absence or presence of 1 $\mu g\ ml^{-1}$ TPA. Incubation was carried out for 5 min at 30°C. The reaction was terminated by the addition of 25% trichloroacetic acid. The acid-precipitated materials were collected on a membrane filter and counted for radioactivity. PKC activity was determined by subtracting the activity measured in the absence of TPA from that measured in its presence and expressed as nmol of $^{32}P$ transferred to histone H1 per min at 30°C per $10^8$ cells. Protein was estimated by the method of Bradford (1976). Bovine serum albumin was used as the standard.

### Results

*Characteristics of TPA-resistant subclones*

The proliferation of MOLT-3 in the presence of 16 nM TPA was reduced with 3 days of culture, after which the cells grew slowly up to day 9, with a viability exceeding 90%, as determined by trypan blue dye exclusion. Thus, the TPA was not toxic to these MOLT-3 cells (Figure 1a). The five TPA-resistant subclones of MOLT-3 obtained in methylcellulose containing TPA grew equally well in suspension cultures, with or without 16 nM TPA (Figure 1b). The form and size of these TPA-resistant subclones did not differ from those of the parental MOLT-3. The resistance of those clones to TPA was not lost for up to several months, even in continuous culture without TPA.

The presence of TdT is characteristic of prothymocytes and is absent in mature T-lymphocytes (Bollum, 1979). The level of the enzyme of MOLT-3 was reduced dramatically in the presence of 16 nM TPA, reaching a level of 12% of the control culture at 3 days, whereas the level of the enzyme of TPA-resistant subclones remained high after TPA stimulation for 3 days (Table I). These results indicate that the parental MOLT-3 reaches a more differentiated state whereas the TPA-resistant subclones remain immature in the presence of TPA.

**Figure 1** Growth curves of MOLT-3 (a) and TPA-resistant subclone (R01) (b) in the presence (●——●) or absence (○——○) of 16 nM TPA. Each point is the mean ± s.e. of viable cell number of two separate experiments. R02, R03, R04 and R05 proliferated equally well, with or without TPA, as R01.

**Table I** Terminal deoxynucleotidyl transferase (TdT) activities of MOLT-3 and TPA-resistant subclones

| Clones | Day 0 | 3 days after TPA stimulation |
|---|---|---|
| MOLT-3 | 60.6 ± 7.8 | 7.1 ± 2.9 |
| R01 | 199 ± 3.2 | 125 ± 9.4 |
| R02 | 106 ± 9.7 | 125 ± 13.4 |
| R03 | 110 ± 7.7 | 106 ± 14.9 |
| R04 | 118 ± 15.7 | 96.7 ± 7.3 |
| R05 | 88.7 ± 14.4 | 70.0 ± 8.7 |

TdT activity: unit per $10^8$ cells. One unit of enzyme activity incorporates in 1 h 1 nmol of dGMP into acid-precipitable material at 37°C, using oligo(dA)$_{12-18}$ as a primer. The values are expressed as mean ± s.e. of three separate experiments.

*Elution profile of cytosolic PKC of MOLT-3 and TPA-resistant subclone R01*

Cytosol preparations of MOLT-3 were fractionated on DEAE-sepharose columns using a linear gradient and column fractions were assayed for PKC activity. The PKC activity eluted at a concentration of 0.06–0.16 M with a peak at 0.10 M NaCl (Figure 2a). Cytosol preparations of R01 showed an elution profile of PKC, in a similar fashion and with a peak at 0.10 M NaCl (Figure 2b). However, the amount of PKC activity in R01 was 40% of that of the parental MOLT-3. The baseline of PKC activity in R01 was lower than that of MOLT-3, thereby indicating that PKC in R01 was not already activated. To determine whether PKC activities of TPA-resistant subclones were less than those of

**Figure 2** PKC activities on DEAE-sepharose chromatography of MOLT-3 (**a**) and TPA-resistant subclone (R01) (**b**). Cytosol from $10^8$ cells (MOLT-3, 10.0 mg; R01, 10.1 mg) were applied to and eluted from the column, as described in **Materials and methods**. PKC activity was determined as described with 50 µl aliquots from the indicated effluent in the presence (●——●) or absence (○——○) of 1 µg ml$^{-1}$ TPA, with 0.1 mM $Ca^{2+}$ and 80 µg ml$^{-1}$ PS and expressed as c.p.m. $^{32}P$ incorporated into histone H1 for 5 min per 50 µl sample. – – – –, NaCl concentration.

**Figure 3** Time course of PKC activities in the cytosolic (**a**) and particulate (**b**) fraction. MOLT-3 (●——●) and R01 (○——○) were incubated for the indicated times at 37°C with 16 nM TPA in 0.0005% DMSO. Each point is the mean ± s.e. of three separate experiments. PKC activity is expressed as nmol $^{32}P$ incorporated into histone H1 per $10^8$ cells.

parental MOLT-3, we assayed the cytosolic PKC activities in MOLT-3 and TPA-resistant subclones by partial purification on DEAE-sepharose columns with a one step with 0.15 M NaCl in 4 ml of buffer A. The amount of cytosolic PKC activity of five TPA-resistant subclones R01, R02, R03, R04 and R05 was 1,171 ± 264 (36%), 1,725 ± 264 (53%), 1,522 ± 119 (47%), 1,689 ± 269 (52%) and 1,634 ± 367 (50%) nmol $^{32}P$ incorporated into histone H1 per min per $10^8$ cells, respectively, all being significantly low compared to 3,247 ± 467 (100%) in the parental MOLT-3. The data are expressed as mean ± s.d., $n = 7$, 7, 4, 4, 4 and 4; MOLT-3, R01, R02, R03, R04 and R05, respectively. Therefore, PKC activities in TPA-resistant subclones were less than that in MOLT-3.

*Effect of TPA on subcellular distribution of PKC*

To investigate the activation of PKC, the effect of TPA on the subcellular distribution of PKC in MOLT-3 and the TPA-resistant subclone R01 was examined (Figure 3). Most (98–99%) of the PKC activity was found in the cytosol and there was little PKC activity in the particulate fraction, in both cell lines. The stimulation with 16 nM TPA in the parental MOLT-3 resulted in a 50% decrease in cytosolic PKC activity within 5 min, followed by a gradual decline to 10% of the initial level at 60 min (Figure 3a) and a concomitant increase in PKC activity in the particulate fraction occurred (Figure 3b). A similar change of subcellular PKC distribution also occurred in R01. Thus, the PKC activities in the cytosol and particulate fraction changed inversely, in both cell lines and in a time dependent manner, indicating that the translocation of PKC from the cytosol to the particulate fraction was caused by TPA stimulation. However, the amount of decrease for 60 min was 0.57 nmol min$^{-1}$ per $10^8$ cells in R01, 24% of 2.40 nmol min$^{-1}$ per $10^8$ cells in MOLT-3 (Figure 3a) and the amount of increase for 60 min was 0.51 nmol min$^{-1}$ per $10^8$ cells in R01, 20% of 2.60 nmol min$^{-1}$ per $10^8$ cells in MOLT-3 (Figure 3b). There was no detectable PKC activity in the cytosol and particulate fractions of both MOLT-3 and R01 at 24, 72 and 120 h after addition of 16 nM TPA (data not shown). DMSO (0.0005%) had no apparent effect on translocation in MOLT-3 or the TPA-resistant subclones (data not shown).

### Discussion

To investigate the role of phorbol ester receptors or PKC in case of TPA-induced differentiation, a comparison between TPA-sensitive and resistant subclones should be of great use. The TPA-resistant subclones from MOLT-3 proliferated in either the presence or absence of TPA. The TPA-resistance was also confirmed by the assay of TdT activities. TdT activities in these subclones remained high even in the presence of TPA, whereas they were reduced in the sensitive parental MOLT-3 cells in 3 days' culture with TPA. These results indicated that the TPA-resistant subclones were not induced to differentiate by TPA and remained immature.

In previous work, we found that the amount of phorbol ester binding to TPA-resistant subclones from MOLT-3 was about half that of the parental MOLT-3, presumably due to a low concentration of receptors for phorbol esters, as assayed by Scatchard analysis (Mayumi *et al.*, 1988). Thus, we concluded that the number of receptors for phorbol esters played an important role in the induction of differentiation in MOLT-3 cells by TPA. Several studies have shown that the receptors for phorbol esters are copurified with PKC (Ashendel *et al.*, 1983; Niedel *et al.*, 1983). The enzyme is now thought to be a major receptor for phorbol esters (Sando & Young, 1983; Sharkey *et al.*, 1984) and also one of the major signal mediators from the cell membrane to the nucleus (Nishizuka, 1984; Berridge, 1984). This prompted us to investigate whether PKC was related to the TPA-induced differentiation in MOLT-3. The levels of PKC in the cytosol in five TPA-resistant subclones were about half those in the parental MOLT-3. A similar result is shown in Figure 2, where the baseline of PKC activity without TPA is also lower in the TPA-resistant subclone R01 than in the parental MOLT-3. These results may reflect a decrease in number of phorbol ester receptors in TPA-resistant subclones of MOLT-3, as already noted (Mayumi *et al.*, 1988).

Phorbol esters with tumour-promoting activity cause a translocation of PKC from the cytosol to the membrane fraction in various cell systems (Kraft *et al.*, 1982; Kraft & Anderson, 1983; Shoji *et al.*, 1987) including human mature T-cells (Isakov *et al.*, 1987; Manger *et al.*, 1987). In cell differentiation systems, however, the role of PKC is not well understood. In the human promyelocytic leukaemia cell line HL-60, Vandenbark *et al.* (1984) found that the TPA-induced differentiation was mediated by PKC, whereas other investigators demonstrated that mediator(s) other than or in addition to the activation of PKC may be required for the induction of differentiation (Kreutter *et al.*, 1985). In contrast, however, the role of PKC in TPA-induced differentiation in T-lymphocytes is less well understood. We found that a translocation of PKC occurred in both TPA-resistant cells and TPA-sensitive MOLT-3 cells as seen in other cells (Kraft *et al.*, 1982; Shoji *et al.*, 1987). However, it should be emphasised that the amount of PKC translocated from the cytosol to the particulate fraction for 60 min in R01 was only 20% of that in the MOLT-3 cells. The amount of cytosolic PKC and of its translocation may be a main factor associated with the induction of cell differentiation.

These results differ from those shown by Homma *et al.* (1986), in that the level of cytosolic PKC in TPA-resistant HL-60 variant cells was as high as that in TPA-sensitive HL-60 cells and that translocation of PKC to the membrane did not occur in the resistant cells. In contrast, the results obtained by Shoji *et al.* (1987), who used the acute myeloblastic leukaemia cell line KG-1, are close to our observations. They showed that cytosolic PKC activity in the TPA-resistant subclone KG-1a was about one third of that in the parental TPA-sensitive KG-1 cells, despite similar patterns of translocation of PKC, in both lines. These discrepancies may be related to different cell systems. With respect to the phorbol ester receptors, there is also a difference between MOLT-3 and HL-60. The down regulation of phorbol ester receptors by TPA was seen both in TPA-resistant cells and in the parental MOLT-3 (Mayumi *et al.*, 1988), whereas it was not observed in TPA-resistant cells from HL-60 (Solanki *et al.*, 1981). The down regulation of phorbol ester receptors was observed within 15 min after stimulation with TPA (Mayumi *et al.*, 1988). The precise relationship between the down regulation of phorbol ester binding and PKC translocation is unclear, but both events may be related.

The role of PKC after the onset of cell differentiation is also unclear. There was no detectable PKC activity in the cytosol and particulate fractions for up to 120 h after treatment with 16 nM TPA, in both MOLT-3 and R01. These results may reflect the continued translocation of PKC from the cytosol to the membrane and the degradation of membrane-associated PKC (Ballester & Rosen, 1985).

While this study provides no direct evidence that PKC translocation from the cytosol to the membrane fraction causes differentiation in MOLT-3, nevertheless, the existence of differences in the amount of cytosolic PKC, the amount of PKC translocation and the number of phorbol ester receptors between TPA-resistant subclones and the parental MOLT-3 cells do suggest that PKC plays an important role in the induction of differentiation by TPA in this T-lymphoblastic cell line MOLT-3.

This work was supported by a Grant-in-Aid for Scientific Research from the Ministry of Education, Science and Culture of Japan (no. 62570291). We thank M. Ohara for pertinent comments.

### References

ANDERSON, N.L., GEMMELL, M.A., COUSSENS, P.M., MURAO, S. & HUBERMAN, E. (1985). Specific protein phosphorylation in human promyelocytic HL-60 leukemia cells susceptible or resistant to induction of cell differentiation by phorbol-12-myristate-13-acetate. *Cancer Res.*, **45**, 4955.

ASHENDEL, C.L., STALLER, J.M. & BOUTWELL, R.K. (1983). Protein kinase activity associated with a phorbol ester receptor purified from mouse brain. *Cancer Res.*, **43**, 4333.

BALLESTER, R. & ROSEN, O.M. (1985). Fate of immunoprecipitatable protein kinase C in $GH_3$ cells treated with phorbol 12-myristate 13-acetate. *J. Biol. Chem.*, **260**, 15194.

BERRIDGE, M.J. (1984). Inositol trisphosphate and diacylglycerol as second messengers. *Biochem. J.*, **220**, 345.

BOLLUM, F.J. (1979). Terminal deoxynucleotidyl transferase as a hematopoietic cell marker. *Blood*, **54**, 1203.

BRADFORD, M.M. (1976). A rapid and sensitive method for the quantitation of microgram quantities of protein utilizing the principle of protein-dye binding. *Anal. Biochem.*, **72**, 248.

DIAMOND, L., O'BRIEN, T.G. & BAIRD, W.M. (1980). Tumor promoters and the mechanism of tumor promotion. *Adv. Cancer Res.*, **32**, 1.

HOMMA, Y., HENNING-CHUBB, C.B. & HUBERMAN, E. (1986). Translocation of protein kinase C in human leukemia cells susceptible or resistant to differentiation induced by phorbol 12-myristate 13-acetate. *Proc. Natl Acad. Sci. USA*, **83**, 7316.

ISAKOV, N., MALLY, M.I., SCHOLZ, W. & ALTMAN, A. (1987). T-lymphocyte activation: the role of protein kinase C and the bifurcating inositol phospholipid signal transduction pathway. *Immunol. Rev.*, **95**, 89.

KAIBUCHI, K., TAKAI, Y., SAWAMURA, M., HOSHIJIMA, M., FUJIKURA, T. & NISHIZUKA, Y. (1983). Synergistic functions of protein phosphorylation and calcium mobilization in platelet activation. *J. Biol. Chem.*, **258**, 6701.

KAJIKAWA, N., KAIBUCHI, K., MATSUBARA, T., KIKKAWA, U., TAKAI, Y. & NISHIZUKA, Y. (1983). A possible role of protein kinase C in signal-induced lysosomal enzyme release. *Biochem. Biophys. Res. Commun.*, **116**, 743.

KIKKAWA, U., TAKAI, Y., MINAKUCHI, R., INOHARA, S. & NISHIZUKA, Y. (1982). Calcium-activated, phospholipid-dependent protein kinase from rat brain. *J. Biol. Chem.*, **257**, 13341.

KRAFT, A.S., ANDERSON, W.B., COOPER, H.L. & SANDO, J.J. (1982). Decrease in cytosolic calcium/phospholipid-dependent protein kinase activity following phorbol ester treatment of EL4 thymoma cells. *J. Biol. Chem.*, **257**, 13193.

KRAFT, A.S. & ANDERSON, W.B. (1983). Phorbol esters increase the amount of $Ca^{2+}$, phospholipid-dependent protein kinase associated with plasma membrane. *Nature*, **301**, 621.

KREUTTER, D., CALDWELL, A.B. & MORIN, M.J. (1985). Dissociation of protein kinase C activation from phorbol ester-induced maturation of HL-60 leukemia cells. *J. Biol. Chem.*, **260**, 5979.

MALAISSE, W.J., LEBRUN, P., HERCHUELZ, A., SENER, A. & MALAISSE-LAGAE, F. (1983). Synergistic effect of a tumor-promoting phorbol ester and a hypoglycemic sulfonylurea upon insulin release. *Endocrinology*, **113**, 1870.

MANGER, B., WEISS, A., IMBODEN, J., LAING, T. & STOBO, J.D. (1987). The role of protein kinase C in transmembrane signaling by the T cell antigen receptor complex. Effects of stimulation with soluble or immobilized CD3 antibodies. *J. Immunol.*, **139**, 2755.

MAYUMI, T., NAGASAWA, K., HORIUCHI, T. & KUSABA, T. (1988). Phorbol ester receptors and the induction of differentiation in the human T lymphoblastic cell line MOLT-3. *Exp. Cell Biol.*, **56**, 12.

NAGASAWA, K. & MAK, T.W. (1980). Phorbol esters induce differentiation in human malignant T lymphoblasts. *Proc. Natl Acad. Sci. USA*, **77**, 2964.

NAGASAWA, K., CHECHIK, B.E., GELFAND, E.W., SENGUPTA, S., LETARTE, M. & MAK, T.W. (1981*a*). Modulation of human T-cell differentiation markers by 12-*O*-tetradecanoylphorbol-13-acetate. *Thymus*, **3**, 307.

NAGASAWA, K., HOWATSON, A. & MAK, T.W. (1981*b*). Induction of human malignant T-lymphoblastic cell lines MOLT-3 and Jurkat by 12-*O*-tetradecanoylphorbol-13-acetate: biochemical, physical, and morphological characterization. *J. Cell. Physiol.*, **109**, 181.

NAGASAWA, K. & MAK, T.W. (1982). Induction of differentiation in human T-lymphoblastic leukemia cell lines by 12-*O*-tetradecanoylphorbol 13-acetate (TPA): studies with monoclonal antibodies to T cells. *Cell. Immunol.*, **71**, 396.

NIEDEL, J.E., KUHN, L.J. & VANDENBARK, G.R. (1983). Phorbol diester receptor copurifies with protein kinase C. *Proc. Natl Acad. Sci. USA*, **80**, 36.

NISHIZUKA, Y. (1984). The role of protein kinase C in cell surface signal transduction and tumour promotion. *Nature*, **308**, 693.

OKAMURA, S., CRANE, F., MESSNER, H.A. & MAK, T.W. (1978). Purification of terminal deoxynucleotidyltransferase by oligonucleotide affinity chromatography. *J. Biol. Chem.*, **253**, 3765.

PARKER, P.J., STABEL, S. & WATERFIELD, M.D. (1984). Purification to homogeneity of protein kinase C from bovine brain-identity with the phorbol ester receptor. *EMBO J.*, **3**, 953.

ROZENGURT, E., RODRIGUEZ-PENA, A., COOMBS, M. & SINNETT-SMITH, J. (1984). Diacylglycerol stimulates DNA synthesis and cell division in mouse 3T3 cells: role of $Ca^{2+}$-sensitive phospholipid-dependent protein kinase. *Proc. Natl Acad. Sci. USA*, **81**, 5748.

SANDO, J.J. & YOUNG, M.C. (1983). Identification of high-affinity phorbol ester receptor in cytosol of EL4 thymoma cells: requirement for calcium, magnesium, and phospholipids. *Proc. Natl Acad. Sci. USA*, **80**, 2642.

SHARKEY, N.A., LEACH, K.L. & BLUMBERG, P.M. (1984). Competitive inhibition by diacylglycerol of specific phorbol ester binding. *Proc. Natl Acad. Sci. USA*, **81**, 607.

SHOJI, M., GIRARD, P.R., CHARP, P.A., KOEFFLER, H.P., VOGLER, W.R. & KUO, J.F. (1987). Effects of phorbol ester on translocation and down-regulation of protein kinase C and phosphorylation of endogenous proteins in human acute myeloid leukemia cell line KG-1 and its phorbol ester-resistant subline KG-1a. *Cancer Res.*, **47**, 6363.

SHOYAB, M. & TODARO, G.J. (1980). Specific high affinity cell membrane receptors for biologically active phorbol and ingenol esters. *Nature*, **288**, 451.

SOLANKI, V., SLAGA, T.J., CALLAHAM, M. & HUBERMAN, E. (1981). Down regulation of specific binding of [20-$^3$H]phorbol 12,13-dibutyrate and phorbol ester-induced differentiation of human promyelocytic leukemia cells. *Proc. Natl Acad. Sci. USA*, **78**, 1722.

VANDENBARK, G.R., KUHN, L.J. & NIEDEL, J.E. (1984). Possible mechanism of phorbol diester-induced maturation of human promyelocytic leukemia cells. Activation of protein kinase C. *J. Clin. Invest.*, **73**, 448.